# Lectures on the Pathological Appearances in the Bodies of the Insane. No. I

**Published:** 1850-04-01

**Authors:** John Hitchman

**Affiliations:** Resident Medical Officer to the Female Department of the Institution


### LECTURES ON THE PATHOLOGICAL APPEARANCES OBSERVED IN THE BODIES OF THE INSANE.

DELIVERED AT THE MIDDLESEX COUNTY LUNATIC ASYLUM,

BY DR. JOHN HITCHMAN,

*Resident Medical Officer to the Female Department of the Institution.*

NO. I.

GENTLEMEN,—Were it not that I feel that this large institution, containing nearly a thousand patients, and situated, moreover, in the metropolitan county of England, should throw open its portals widely to all those who seek for information in the most difficult and most important of diseases; were it not, that I as strongly feel that its medical officers, however feeble their talents for observation may be, or however weak their power to enunciate the result of these observations, should, nevertheless, contribute their "little all" to enlarge the boundaries, to increase the resources, and to elevate the aims of this department of science, I should have shrunk from addressing you. And I should have done this all the more readily, because our able and distinguished physician has so fully set before you the symptoms and treatment of the malady, and because his extensive experience, his great acquirements, his elegant diction, and didactic skill, make it appear something like presumption in me to occupy the place which he has so recently vacated. I *know,* however, that with him, and I trust also with you, the will to act will be received with favour, and that my humble efforts to advance this great cause will neither be misunderstood nor unappreciated. The task which has been assigned to me is, to state the facts which have come before me in the autopsy-room of this great institution, and to draw from them such inferences as may appear to me consistent with the established laws of physiology, and the rigid requirements of a purely inductive science.

Thirty-seven patients have died in the female department of the Asylum, between January 1st, 1848, and the commencement of this course of lectures. Their respective ages, divided into decennial periods, were as follow:—

| | |
|---|---|
| Between 20 years and 30 | 4 |
| " 30 years and 40 | 6 |
| " 40 years and 50 | 7 |
| " 50 years and 60 | 5 |
| " 60 years and 70 | 7 |
| " 70 years and 80 | 4 |
| | 33 |

There were four patients of whose ages there was no record; of these, two were apparently between the ages of thirty and forty years; one between forty and fifty years, and one between fifty and sixty years. The *duration* of the mental disease in seven of these thirty-seven cases was unascertained. Of the remaining thirty, two had been insane for a period not exceeding two months; two for a period not exceeding one year; one for nearly two years; three for nearly three years, and two for nearly five years. But to avoid being extremely tedious, I will arrange the remaining twenty in quinquennial periods.

| | | |
|---|---|---|
| Between | 5 and 10 years | 9 |
| „ | 10 and 15 years | 2 |
| „ | 15 and 20 years | 4 |
| „ | 20 and 25 years | 4 |
| „ | 25 and 30 years | 1 |

The above cases were classified as suffering respectively from the following forms of mental disease:—

| | |
|---|---|
| Mania | 8 |
| Mania, with General Paralysis | 2 |
| Mania, with Epilepsy | 1 |
| Melancholia | 1 |
| Convalescent from Melancholia at the time of her decease from Pneumonia | 1 |
| Imbecility | 10 |
| Senile Imbecility | 2 |
| Imbecility, with Epilepsy | 3 |
| Imbecility, with General Paralysis | 3 |
| Dementia | 2 |
| Dementia, with General Paralysis | 1 |
| Dementia, with Epilepsy | 3 |
| | 37 |

Of the above patients, two died in the acute stage of mania: one in one month after the attack, in the sixty-seventh year of her age; the other, aged thirty-six years, had been deranged in her mind nearly two years, but died from exhaustion consequent upon recurrent maniacal excitement. Six of the above patients died from "general paralysis:" in one of these, the paralysis was associated with pulmonary consumption, and in another with abscess about the parotid gland; only one of these patients had reached the age of forty years: the average age of the six being thirty-three years. Four died from "apoplexy;" three of these were the subjects of epilepsy; the apoplexy was of the congestive kind in two out of the four, and was probably the consequence of epilepsy; in the other two there was extravasation of blood, in one, upon the cranial surface of the dura mater, and in the other, into the left cerebral hemisphere, and was the consequence of a previous softening of that structure. These apoplectic cases had respectively attained the age of forty-seven, fifty-five, forty-eight, and sixty-one years. One died in a fit of "epilepsy" (age unknown); one in the sixtieth year of her age, from "paralysis agitans." Of the remaining twenty patients, there died, from "pulmonary consumption," one; from "pneumonia" and its consequences, five; from "pericarditis," one; from "fatty degeneration" of the heart, one; from "hydrothorax," one; from "scirrhus of the liver," one; from "cirrhosis" of that organ, one; from "inflammation of the bowels," two; from "erysipelas," one; from "cancer of the breast," one; from "dysentery," one. One was brought into the Asylum in all but a moribund condition, whether from fever or mania, I could not satisfactorily ascertain: one died from the "general decay" of old age; and the remaining five were classed under the head of "general debility." This last term "general debility" has been applied by Dr. Conolly to "cases in which the performance of every function seems to become enfeebled by long continued disorder of the nervous system." You will observe that in this catalogue of the causes of death, fourteen belong to the class neuroses, or diseases directly involving the nervous system; exhaustion after mania, two; "general paralysis," six; "apoplexy," four; "epilepsy," one; "paralysis agitans," one: making, in fact, nineteen, if we add the five caused by "general debility." Even fourteen cases out of thirty-seven, as it is considerably more than one-third of the whole number, prompts the question — Has insanity a tendency to shorten the duration of human life? It would seem that this is a question which statistics alone can answer, but figures are among the most deceitful things in the world, and many circumstances must be taken into consideration before we can arrive at accurate results,—for instance, a short time ago, "the mortality in prisons was calculated by dividing the number of committals by the deaths." The result thus obtained was so favourable to the healthiness of prisons, as to have led a French minister to pronounce prisons the healthiest places in the world: and an English inspector gravely to affirm, "that in very few situations in life is an adult less likely to die, than in a well-conducted English prison." Only one in five hundred prisoners die; so, according to this view, Mr. Farr says, "If a man desire to live to the age of Methuselah, he should go to Newgate."—(Dr. Seller, *Edin. Med. and Surg. Journal*, Oct., 1848.) This calculation overlooked the fact, that the inmates of a prison are renewed, on an average, eight times in the year, so that if one in five hundred be the mortality for the eight-part of a year, it represents a mortality of sixteen per thousand, which is more than one-half greater than that of the population in general, at the average age of prisoners. The mortality in this Asylum, during the past ten years, has been 7.78 per cent.; and I find from a most valuable article on Lunatic Asylums, in the Supplement of the *Penny Cyclopædia*, that it has, in the Norfolk Asylum, from 1836 to 1845, reached as high as 19.74 per cent.; and at Lancaster, 14.94 per cent.; and Stafford nearly as high; the mean of 44 Asylums in England, Ireland, and Wales being about 9.62 per cent. There are not yet observations enough made on an accurate basis to determine the mortality of lunatics at different ages, but assuming, as Mr. Farr has done, that the mean age of lunatics does not *exceed* forty to forty-five years; then, if, according to Quetelet, the average mortality of the population at that age be 1.17 per cent., or, according to Farr, 1.50, it follows that insanity, to use Dr. Farr's words, "*increases the mortality six-fold.*" This calculation gives such an excess of mortality, so to speak, as completely to set aside any difference which may ensue from a slight error in the probable average age of the lunatics referred to, in making a comparison of the mortality of the insane and sane. Again, a tolerably extensive experience among the insane, enables me to state that, with the exception of fever, there is no disease, which they are not as liable to as the general population, while they incur the additional risks of the affections incident to mental derangement; and therefore, with all these facts before you, you may positively affirm that insanity *has* a tendency to shorten the duration of human life; nay, that it increases the mortality at least threefold. I say threefold, because there are some circumstances, not taken into consideration by Mr. Farr, which tend to diminish his high estimate; for instance, when the lunatic has passed through the malady for several years, and arrived at the age of sixty years, there is not so great a difference between the mortality of that age among lunatics, and among the general population, as at the period of life selected by Mr. Farr for his calculations and inferences. I have been minute, and, I fear, tedious, in these details, because great mistakes still prevail among medical men on this subject, and I am frequently asked by the public, whether insanity does not tend to longevity? Dr. Burrows, in his "Commentaries on Insanity," undertakes to deny this latter supposition, but demurs as to whether it directly shortens existence. I gave the class of last year an account of a law-suit on this very topic, in which, in consequence of the discrepancy among medical men, as to whether insanity had, or had not, a tendency to shorten human life, the majority having come to the latter conclusion, a Life Insurance had to pay over (we now know how unjustly) to the executors of a lunatic, the sum of 2,000*l.* The weekly medical press of the period espoused the opinion of the majority, and thus perpetuated, for awhile, the error, and the wrong arising out of it. We can readily perceive the data from which such erroneous opinions spring, even in the small number of thirty-seven patients, whose deaths we have brought before you; *e. g.*, four patients had reached a good old age, seventy-two, seventy-four, seventy-five, and seventy-six, and one of these had been insane twenty-eight years. A few isolated facts, such as the great age of an individual lunatic, like to some of those you have seen in No. 2 Ward, or an obituary limited to three or four persons who may have died in his neighbourhood, has ever enabled the observer, provided he be a "practical man," and no mere "theorist" or vulgar "statistician," to come to positive conclusions. Still those who are less gifted with the egotism of "practical experience," and therefore more cautious, find it necessary in all such matters as these to search over a large number of facts, and over a great space of time, and thereby learn that insanity does shorten the duration of human life; that the mortality of the insane is greater in the earlier stages of the malady than in its subsequent stages; and that it is more fatal among the male than among the female patients. It is in the matter of births and deaths, or such like positive facts, that statistics are so valuable. In reference to the comparative merits of different modes of treatment, or the special fatality of certain diseases, I have less confidence in them, inasmuch as these must be so much influenced by the severity of the malady, on the one hand, and the diagnostic skill and therapeutic knowledge of the practitioner, on the other, that no precisely accurate conclusions can be deduced from them. And this reminds me to state, that in assigning the causes of death in the above thirty-seven cases, we have fixed upon the most conspicuous and serious lesion. Nature defies the simple classification of the nosologist; and in the examination of several hundred bodies, I have rarely found disease to be limited to one organ, much less to one structure; for example, the case marked as having died from "pericarditis," had also hepatization of the upper lobe of the right lung, and pleuritic adhesions over it, and over the same lobe of the left lung, together with minor changes in other organs of the body. There is one feature in the table of causes of death which requires a passing remark, otherwise you may be led into a fatal inference from the unusual circumstance of there being only one recorded case of pulmonary consumption, out of thirty-seven deaths, or, strictly speaking, two; one being associated with general paralysis. In the year 1846, eight deaths out of twenty-four were from this cause; and in 1847, six deaths out of twenty-one: and from facts in my possession, extending over a series of seven years, I should say, that more than 20 per cent. of the deaths among the female insane arise from pulmonary phthisis, and from a disease which has been termed by Andral chronic pneumonia. It is an affection in which there seems a union of pneumonic induration and grey tubercular infiltration. The lung under such circumstances is so heavy, that it falls like a stone when dropped into water, and the bronchial twigs are choked with a white chalk-like substance; the colour of the lung resembles the appearance of granite, with occasionally stripes of white colour across the cut surfaces. There are in the upper parts of the lung some small cavities containing pus. It will be found, moreover, that the *tendency* to pulmonary consumption is nearly as great among the male insane. If the calculation be made upon the *number of deaths*, this will not appear, because of the great mortality which is caused by general paralysis; but if the deaths from phthisis be calculated upon the annual number resident, through a series of years, this approximation will be recognised. I must, however, add, that there are no published records upon an extensive scale in England, from which you can draw safe inferences in this matter; because hundreds of lunatics have died with tuberculous lungs without the fact having been recognised, so completely has the pulmonary affection been modified and concealed by the mental derangement. Leaving, however, the statistics of this interesting subject, I must inform you that insanity greatly obscures the manifestations of bodily disease. In the wild excitement of acute mania, or in the profound distress which characterises some cases of melancholia, this may have been expected, nay, it has been recognised, and beautifully illustrated by our revered dramatist, (or as I may perhaps have here called him, the great psychologist, Shakspeare,) where he makes the unhappy king exclaim—

> "Where the greater malady is fixed,
> The lesser is scarce felt. Thou'dst shun a bear;
> But if thy flight lay toward the raging sea,
> Thou'dst meet the bear i'th the mouth. When the mind's free,
> The body's delicate: the tempest in my mind
> Doth from my senses take all feeling else
> Save what beats there."

But it is not in these forms of insanity alone that such influence is exerted over bodily derangements. In chronic mania and imbecility, we observe the same influence, and especially in their power to modify and conceal pulmonary diseases. If life be pleasing to the lunatic,—and we who are in daily communication with them know that it is,—then have they of all persons reason to be thankful that the genius of Laennec has taught us to recognise the whisperings and murmurs of disease, even where no vital phenomena or symptoms have displayed its existence; indeed, the psychologist must be attentive to physical diagnosis, not only as regards the pulmonary organs, but all others, if he is to bring relief to those under his care. Dr. John Davy having recognised the fact to which I am now alluding,—namely, that pulmonary consumption may run its fatal career in the insane without any cough or expectoration, and stating, that there was in such cases a sensible increase of temperature under the tongue, I had this little thermometer made to ascertain the existence of this phenomenon; but it is so difficult of application in many cases, and so liable to error from the manner in which it may be embraced by the lips and tongue, that I have ceased to use it, and rely wholly on percussion and auscultation, which seldom fail to recognise the malady. Cases do, however, occasionally occur, in which the general health has been such as not to rouse suspicion until the disease has reached a very advanced stage. Thus, J. G., an epileptic patient, who died during the past year, became very ill on Tuesday, January 16th, with vomiting, accompanied by much febrile disturbance and some slight diarrhœa. On percussing the chest, I found extreme dulness in the supra-clavicular and supra-scapular regions, with a perfect absence of the respiratory murmur; in other parts, pectoriloquy was well marked; while throughout the lung there were unequivocal signs of extensive organic change. On the 22nd, six days from the first appearance of illness, she died; and in the post-mortem examination the chest presented the following appearances:—The right and left lungs bound down by pleuritic adhesions; the apex of the left lobe indurated, impervious to air, and strewed with granular matter; in the lower part of the upper lobe there were several tubercular cavities, while the lower lobe was disintegrated, having a large abscess in its interior, which had escaped into the cavity of the thorax. The right lung was also tuberculous, but not nearly to the same extent.

Others die having scirrhus of the ovaries, or of the mamma, and not a murmur escapes them of pain or uneasiness; others so blend up their bodily sufferings with their mental hallucinations, that your attention is directed to their bodily ailments by the peculiar character of their delusions. Dr. Conolly has given you some illustrations. One poor woman, E. J., who died in January last from chronic peritonitis and inflammation of the bowels, used to state that I cut her with knives over the abdomen during the night, and that I employed invisible devils to do it during the day. This woman evidently felt great pain occasionally, but still she pursued her ordinary avocations about the ward, cleaning, and the like, until a few days before her death. In the phthisical cases, and in many others, there is not the same secretion, irritation, and pain as are felt by the sane under similar diseases. Emaciation goes on rapidly—turbercle is evidently suppurating in the lungs, and yet no cough, no expectoration proclaim the fact! How is this? Did it take place in every case;—if all lunatics who were phthisical were devoid of cough and expectoration, one might imagine that there was some truth in the supposition entertained by Mr. Rainey, of St. Thomas's Hospital, that the arachnoid membrane was, in fact, a membranous plexus of organic nerves; and thence infer that by its diseased condition it implicated the organic nerves throughout the system; but as some lunatics are exempt from this anomaly, whose cerebral meninges are equally diseased with others who display the above peculiarity, this supposition will not hold good; and again, it only explains the absence of secretion and irritation, but not of pain. Still, that the condition of the arachnoid membrane, and of the vesicular neurine of the brain which it covers, do influence the symptoms of disease in other organs, is unquestionable. A pulmonary patient gasping for breath, and unable to speak a sentence, becomes delirious; and from that moment he shouts and vociferates so long as the delirium lasts, as though his lungs were free from disease; and I have shown you, by the autopsy of J. G., (and I could have added fifty others,) how silently such diseases proceed to their fatal termination, so far as cough and expectoration are concerned, even in the *chronic* diseases of the cerebral organ. Pathology, studied carefully, with a close comparison of the symptoms observed during life, will aid the science of physiology, and reveal more of the true functions of the various parts of the brain, than all the vivisections which have been so skilfully performed by Magendie, Flourens, and others; but these remarks are foreign to our purpose.

Of the eight cases of mania, unassociated with epilepsy or general paralysis, two died while labouring under the malady in its acute form; in one of these the disease was recurrent, and occurred in a female who had been insane nearly two years; in the other, the insanity was quite recent, not having exceeded five weeks' duration. This patient, whose autopsy we shall now describe, was in the sixty-seventh year of her age. The form of the skull was normal; it was free from disease, and rather more than one-eighth of an inch in thickness.

The dura mater was firmly adherent to it; this membrane was inflamed, and the superior longitudinal sinus was large, having a gritty, bone-like deposit upon its exterior surface, and long fibrous bands extending to about half-an-inch on each side of it. The arachnoid membrane was slightly inflamed, but not thickened; there were about three drachms of red-coloured serum beneath it, but this was limited to one spot, and served to fill up a hollow in the upper surface of the anterior cerebral lobes, to be presently noticed. The pia mater was greatly congested, giving to the convolutions on the superior and lateral parts of the cerebral lobe, a scarlet, arborescent appearance. The encephalon weighed two pounds eight ounces avoirdupois. It was well formed, except that in the upper part of both cerebral lobes, at a point subjacent to that part of the skull which the phrenologists have marked out for the organ of "Veneration," or rather at the junction of this faculty with the organ of "Benevolence," there was a cup-like cavity formed by the junction of the two lobes, and caused by an atrophied state of the convolutions, capable of containing three or four drachms of fluid. As we have before observed, the calvarium itself was smooth and regular in form, and presented no corresponding depression. The convolutions at this spot were shrunken and atrophied laterally, as well as in depth; they were slightly softened at this place, possibly from the fluid resting upon it. (I believe this to have been a post-mortem change.) In other parts they were firm; the vesicular neurine, or "grey matter," of the convolutions, was a pink or rose colour, more intense in its hue in the anterior and upper convolutions of the brain than in the posterior or inferior lobes; the three layers into which the "grey matter" seems to be divided were well marked, owing to their variations in colour, the middle line being more scarlet in its hue, and rendering the division as distinct in the convolutions of the upper surface of the anterior cerebral lobe by its rosy tint, as it at all times is in some of the convolutions near the posterior cornu of the lateral ventricles, by the white, thread-like film of matter which traverses them; the laminæ of the cerebellum were not so highly inflamed as the neurine of the cerebrum; the difference was well marked; the white or fibrous matter of the cerebrum was much congested, presenting many bleeding spots when sliced; the ventricles of the brain contained about a drachm of colourless fluid; the corpora striata were much injected, as were the optic thalami and the choroid plexuses; the lungs were free from disease; the pericardium was adherent to the anterior surface of the heart, and was separated with extreme difficulty, and not without lacerating the muscular fibres; there was no fluid in the sac; the muscular structure of the heart was soft; the right auricle was greatly dilated, and its internal lining inflamed; the tricuspid valves were greatly thickened, this fibrinous concretion extending to the tendinous chords, and rendering them thick and knotty, and even reached upwards, involving the auricular lining; the mitral valves were thickened, but not to the same extent; strong marks of endocarditis were visible in both ventricles; the stomach and liver were healthy, but the mucous lining of the small intestines was greatly congested and inflamed. In the other patient, who died in the acute stage of mania, there happened to be a similar condition of the heart, in a lesser degree, accompanied with great dilatation of the pulmonary artery a little above the semilunar valves.

The next autopsy which I shall select for your notice is that on the body of S. F., a patient who was seventy-five years of age, who had been deranged in her mind twenty-seven years, eighteen of which had been passed in this Asylum. She was unable to identify persons with accuracy, and imagined she had seen parties before, and assigned to them a history, of whom in reality she knew nothing, and whom, in fact, she had never seen prior to the moment of addressing them; she was garrulous and somewhat suspicious, becoming angry if her name was mentioned in conversation by other parties. About two months prior to her decease, she became more excited than she had been for several years—was restless at night, walked about the room, pulled off the clothes from the beds of other patients, and wandered about as if in search of some imaginary object or person; this state of feeling passed away after the exhibition of a few doses of acetate of morphia. She died from pericarditis. In the autopsy we found that the skull was normal in thickness and moderately well formed; the dura mater healthy; the arachnoid membrane was beautifully transparent, and the brain could be distinctly seen in some parts a quarter of an inch below it, the interspace being occupied by a clear serum, translucent as spring water; the veins ramifying on the arachnoid were large and of a very deep blue colour; small veinlets could be distinctly seen passing from the convolutions through the fluid to the under surface of the membrane; and it was interesting to observe the minute threads of other blood-vessels passing from a rich brown colour to a beautiful scarlet hue from the action of the atmosphere. When the dura mater was first removed from the brain, these small vessels to which I refer were resting on the face of the convolutions, and were of the colour of a ripe chestnut, but in less than five minutes they became of a bright red; the transition was gradual and complete. I mention it, because it assumes an importance in a pathological point of view, it being *a post-mortem* change, and likely to mislead a person (who might see the brain for the first time five minutes after the membrane was removed) by indicating an amount of arterial excitement which did not exist. The clear serous fluid embraced the whole encephalon, but was most abundant over the superior portions of the anterior cerebral lobes; the membrane when removed was very slightly, if at all thickened, and there was not an opaque spot upon it except immediately in the neighbourhood of the Pacchionian bodies, which latter were slightly enlarged. The grey matter, which I will hereafter call the vesicular neurine of the convolutions, was pale and soft; the other parts of the encephalon were healthy. I should have stated that it weighed three pounds avoirdupois. The lungs were hepatized (grey induration) at their *apices*, and sunk immediately on being placed in water; the heart was hypertrophied, more especially the left ventricle; it weighed eleven ounces, was covered with fibrinous lymph, and immersed in a thick fluid of a greenish hue, having small shreds of the same matter which covered the exterior of the heart floating in it; the valves were healthy. There was no important lesion observed in the other parts of the body. The great quantity of fluid surrounding the brain would lead to the belief that the patient died from serous apoplexy—the symptoms during life did not indicate this; and great quantities of fluid are found in the brains of lunatics who have been long in a state of imbecility, and whose brains are shrunken. It is *conservative* in its nature, and is invariably found to exist in an inverse ratio with the size of the brain as compared with the cranium; it is sometimes local, filling up the sulci of atrophied convolutions, or occupying a cavity or depression in the surface of the encephalon, as in the case of S. O. I always look as a matter of course for its presence in chronic cases of dementia, from whatever cause they may have died.

C. L., aged fifty-four, had been insane twenty-two years. Chronic mania.—Calvarium very thick; dura mater firmly adherent to it; arachnoid membrane white, opaque, thickened, capable of being torn from the surface of the brain with ease; pia mater much congested; serum between the gyri of the convolutions; brain pale, and somewhat soft; lateral ventricles containing about an ounce of fluid; lungs bound down to the chest by old pleuritic adhesions; other structures healthy. This is a fair type of the cerebral appearances in chronic mania.

We will now pass on to an illustration of one of the most important complications of insanity that can possibly fall under your notice—namely, that of general paralysis. Its symptoms have been portrayed to you by a master hand, and I feel assured that, after the many illustrations of the disease which you have seen in the wards, and after the graphic description of its commencement, progress, and termination, which you have listened to, you will readily recognise it, if it should hereafter come before your notice as practitioners.

Six cases, suffering under general paralysis, have died during the past fifteen months. They presented several anomalies—not one lived eigtheen months after the attack became evident; one, not six months. Four out of the six were morose, sullen, gloomy, and unhappy; and only one exhibited the hopefulness, the buoyancy, the elated self-satisfaction, which usually characterise the malady among the male patients. Only one, C. K., out of the six, possessed the ample forehead, the well-formed, full-sized anterior cerebral lobes, which have frequently been observed, in this Asylum, to co-exist with general paralysis; in only one brain was there a generally diffused hardness of structure, and strong tenacity of fibre, resembling a brain hardened by alcohol, which characterised the cerebra of the female paralytics which I examined during the year 1846 and part of 1847, and which is common among the male patients. So strong does the fibrous matter of the brain become, that I have seen the greater part of the organ lifted up from the table, by seizing on the upper portion of the septum lucidum, and this normally delicate structure remain unlacerated by the attempt; still there was, in a few cases, a firm abnormal consistence of the medullary matter; and the lining membrane of the lateral ventricles of the brain was strong, tough, and gritty to the touch, resembling finely pulverized glass upon paper. In every case, there was a thickened arachnoid membrane, a pia mater congested with venous-coloured blood, and considerable effusion of serum beneath it, and beneath and within the sac of the serous membrane, as also in the lateral ventricles. In one case of the six, there was softening of the medullary matter of the brain, a condition which I have found, on former occasions, in similar affections, and which has been recognised by all observers in this malady—in this, as in every case in which I have found a soft, almost pultaceous, condition of the peripherean surfaces, and the white fibrous matter of the brain, the general paralysis had existed a long time; the patient had lost all power of locomotion, and even of prehension; the sphincters had long been useless; the legs were contracted, and drawn up towards the chest; the skin had lost its vitality in many parts, and sensation was blunted over the general surface, and wholly lost in the lower extremities; such patients were also in the lowest stage of dementia. The six cases differed so little in the pathological appearances of the brain, that I shall limit myself to the detail of one case, as the type of the other five, which I select from no especial peculiarities, but because it was the last case of general paralysis which has occurred on the female side of the asylum. Her name was S. B.; she was about forty years of age, and had been insane six months. The mental derangement was ascribed to conjugal strife. Insanity is hereditary in her family, and she was the sister of L. B., a dangerous lunatic, now in Number 8 Ward. The symptoms of general paralysis were, in all probability, coeval with the mental disease; for on her admission, twelve weeks after the attack of insanity, there was great tremor about the lips; the tongue was protruded slowly, and with that quivering propulsory effort, to which your attention has been frequently drawn. Her speech was deeply involved; she walked with extreme difficulty, and could not carry anything steadily to her mouth with her hands. She was restless and noisy, but still good-tempered, during the twelve weeks she lived in the Asylum. She died on the 10th of December, 1848.

The calvarium was thin; the dura mater adherent to its under surface; the arachnoid membrane was thickened and opaque, containing serum within its sac; there was also a considerable quantity of effused fluid beneath it, and also under the pia mater, filling up the gyri of the convolutions. The cineritious neurine was somewhat soft; the fibrous matter was strong and shining. The foramen occupying the anterior commissure was so large, patulous, and firm, that I could place the point of my little finger in it without laceration.

The ventricles contained about one ounce and a half of clear fluid; their lining membrane was strong and gritty to the touch. The lungs were tuberculous, and the heart presented unequivocal traces of endocarditis. These changes in the brain are so like those commonly met with in the brains of lunatics, that the description of one seems almost the echo of all others; but in these cases of general paralysis, there is superadded to the appearances found in mania uncomplicated with this dire affliction, the hardness of the medullary fibre, and the gritty condition of the ventricular lining; still, even this latter particular has again and again been found in cases which were free from general paralysis, while in others, as I have before named to you, there is the general softening of the whole medullary structure. I also remember having seen the brain of a male patient, in whom there was no lesion to be discovered at all characteristic of general paralysis; in a second, I recollect, that while the upper surfaces of the brain were free from any apparent disease, there were great hardness and a shrunken condition of the pons Varolii and medulla oblongata—these latter appearances of course fully accounting for any amount of impaired action in the voluntary muscles. I have carefully examined the spinal chord in two cases of general paralysis—in one I found it had undergone considerable change in various places, especially in the cervical portion, and the canal contained a great quantity of fluid; in the other case, there was no appreciable lesion; the only peculiarity was, that the chord did not extend so far down the spinal column as it is ordinarily found to do—*i. e.*, it did not reach the first lumbar vertebra, but terminated opposite to the inferior edge of the eleventh dorsal vertebra. In this patient there was an entire loss of power of the lower extremities, accompanied by great contraction of the flexor muscles.

It now becomes my duty to tell you that the precise pathology of this affection is involved in much obscurity. It has received but little attention in England—so little, indeed, as to induce Dr. Burrows to think that the disease is more common in France than with us. I believe it to have been long overlooked: in my early career I saw many such cases, but did not know the affection by the term "general paralysis." I have recognised its presence in every asylum I have visited, except Bethlehem or St. Luke's, from which hospitals it is especially excluded. The first writer on its pathology was Monsieur Bayle, in 1822, who ascribes the disease to chronic meningitis. Calmeil, who published his work in 1826, ascribes it to a softening of the cineritious neurine. The latest author is Monsieur Rodriguez, professor of medicine at Montpelier, who has published his work so late as 1847. An analytical review of this book has been given by Dr. Winn, of Truro, in the *Psychological Journal,* and published subsequently as a distinct work: it is from its pages that I have gathered the experience and opinions of the French author. This gentleman has given us the results of twenty-one dissections. Congestion of the scalp; variations in the form, and thickness, and character of the bones of the skull; induration, thickening, and adherence of the dura mater to the calvarium; changes in the character, and effusions beneath and within the arachnoid membrane; serous infiltration of the cellular tissue of the pia mater; hyperæmic and anæmic states of the brain, and softening and induration of this organ, with effusion into the lateral ventricles, are the changes which occurred in some one or other of the cases. There is one passage to which I solicit your especial attention: it is this—"Hardening of the brain occurred as frequently as softening, and was accompanied by either hyperæmia or anæmia. In some cases the cortical substance and the ventricular walls were *indurated,* the other parts, nevertheless, being in a normal state. In one case, *softening of the superficial parts of the brain occurred,* whilst the *deep-seated portions were hardened.* In one case, cited from Calmeil's work, in which the skull had sustained a fracture, with loss of substance of the parietal bone, the part of the convolutions contiguous to the bony aperture was firm and resisting, and covered a layer of hardened brain. In another instance, the colour of the hardened brain varied, from a light coffee colour on the outside of the brain, to a reddish tint on the inside. The cerebellum and *pons Varolii were indurated in one case.*"—p. 356. M. Rodriguez asserts "that all alterations produced by inflammation begin with softening, and end with hardening;" and his opinion of the true pathology of this formidable malady is thus given: "The disease commences with sanguineous congestion, which is discoverable by symptoms differing in intensity, and which may be occasioned by a blow, or some other cause. This congestion," he says, "gives rise to chronic inflammation of the membranes, and cerebral disturbance, indicated by delirium and agitation; the inflamed arachnoid, in the next place, pours forth serum upon the surface of the brain, into the ventricles at the base of the brain, and into the vertebral canal, producing general paralysis."

In support of his opinion that meningitis is the cause of "general paralysis," the author quotes M. Bayle, who gives fifty cases of general paralysis, in which he found chronic meningitis. (I have never seen one case of general paralysis without it.) Chiarugi, of Florence, Neumann, of Berlin, the great name of Esquirol, and many other authorities, are brought forward to support these facts and doctrine. In some of M. Calmeil's cases, these membranes were partly fibro-cartilaginous; and M. Rodriguez gives an instance where "an effusion between the dura mater and the external layer of the arachnoid membrane had completely dissected that membrane from the great falx, to the tempero-parietuo suture, and from the coronal fossa to the posterior edge of the parietal bones on each side." "There was sometimes found an albuminous effusion upon the arachnoid membrane, which had some analogy to trembling jelly."

I dissent from this writer's conclusion, but I accept his facts with thankfulness, for I recognise their truth; and, singular enough, while the ink which I used to abstract the notes was yet wet on the paper, a case, closely resembling the last detailed, fell under my notice. It was a male patient, H. R., and Dr. Begley invited me to look at what he properly regarded as an unusual circumstance—the arachnoid membrane on the left side of the brain (and it was limited to that side,) had acquired the thickness of fibro-cartilage, and was covered over with a soft yellow-coloured lymph—the patient had died from apoplexy, while suffering from general paralysis. I regarded this thickening as the result of previous inflammation, and the lymph which was diffused over its surface, as the remains of effused blood, it being of the same colour and character (though somewhat softer in consistence) as the fibrinous, yellowish-coloured clots, which are so commonly found after death in the ventricles of the heart. I did not consider this thickened membrane as the cause of the general paralysis, but felt assured that the man must have been apoplectic on a former occasion, and that he had suffered from hemiplegia of the right side. I told Dr. Begley that I suspected the patient must have been hemiplegic prior to admission, and he kindly placed before me a very full account of the patient's history, in which I found my suppositions verified. A past hemiplegia may, or may not, have been the state of things in M. Rodriguez' special case. But to pass to his conclusions respecting the general opacity and disease of the membranes. It does not follow, that because Tenterden-steeple and the Goodwin Sands are found together, that they must be regarded as cause and effect; and so with the thickened membranes and general paralysis: they are perhaps associated in ninety cases out of one hundred; but then, membranes as thick, as strong, and as opalescent are found in the same number of cases, who have never exhibited the slightest trace of general paralysis. It is exceedingly rare to find a *healthy* arachnoid membrane upon the brain of a lunatic who has been insane two or three years; and inasmuch as general paralysis frequently extends over two years, and is during the whole of that time associated with mental derangement, a diseased condition of the serous membrane may always be expected, as coincident with, but not the cause of, general paralysis. We must, in investigating the pathological characters of general paralysis, rid the subject of superfluities, and thus be in a condition to see and appreciate some one especial case which shall elucidate its true nature. In the first lecture which I had the honour to deliver in this room, I stated that "the pathology of insanity, as described in books, is most unsatisfactory; and if ever a perfect light be thrown upon this truly mysterious subject, it will be, in all probability, by the examination of some single case, marked with prominent peculiarities: for example, from the dissection of the brain of some patient who had exhibited intellectual delusions, unaccompanied by paralysis or epilepsy, and who had been deprived of life by an accident, not interfering with the condition of the brain, and fortunately falling under the examination of some person accustomed to reflection, and acquainted with the assemblage of appearances commonly found in the brains of lunatics"—in other and better words, from what Bacon calls, some "glaring instance." So with general paralysis, its pathology will be discovered from some single instance; but the discoverer will have been previously experienced in the post-mortem appearances common to such cases, either by the recorded experience of others, or by his own; in this respect, at least, I hope to be useful. First, then, the thickened membranes may be removed from the list of assigned special causes of general paralysis, for I have seen them in two hundred cases in which there was no general paralysis. Different conditions of the pia mater, and tumours of the cerebrum, have also been recorded in the history of these diseases—the former being strewed with hydatids, in one case. The position and character of the tumours will determine the influence they may have in producing this malady; but I have seen the pia mater, in a case hereafter to be described, strewed with these parasites, and the patient was free from general paralysis. In one case of this disease, M. Rodriguez found the spinal marrow compressed to the thickness of a riband by a tumour; and Professor Carswell has given one or two beautiful illustrations of disease of this structure in similar affections. In Carswell's cases, there is a yellow discoloration on the side of the upper part of the spinal chord, and this alteration of structure was found to reach the grey matter in the centre of the chord. These are most instructive cases, and merit our greatest consideration. And here let me state, that if I have dissented from the conclusions of M. Rodriguez, or, indeed, from any other labourer in this great and difficult field of research, I do so in no flippant spirit, or with any censorious feeling; on the contrary, I reverence them for their research—I feel grateful for the result of their labours, and for the suggestions which they have given respecting the nature of this disease; their opinions, when adverse to my own, make me re-examine my facts and my conclusions; but no true son of science, no earnest lover of the truth, would wish me to forego the teachings of nature, or to conceal the convictions of my own mind.

It is in this spirit that I have just closed the pages of Monsieur Calmeil's great work—a work which merits all the eulogies which Dr. Conolly has bestowed upon it; and I am sure Calmeil would not seek for higher praise; Calmeil's facts are invaluable, but it is my misfortune to differ from his conclusions. He states, that the disease is a phlegmesia, and that its seat is *in the cineritious neurine* of the hemispheres. I could have believed in this opinion, had I never seen an inflamed peripheral surface unassociated with general paralysis; but I have seen this structure in a state of the most intense inflammation, where there has been none of the signs of the malady in question; and in the person of S. B., who died in this Asylum, having a large fungoid tumour, arising from the dura mater, the anterior and middle lobes of the left cerebral hemispheres were in a state of pulp throughout their peripheral surface, with none of the pathognomonic symptoms of general paralysis. Monsieur Foville ascribes the disease to an adhesion and hardness between the separate planes of the medullary fibres; while to add to the general difficulty, Monsieur Lelut, in the Annals of Medical Psychology, has published two cases of general paralysis with dementia, in which there was no lesion whatever in the brain or spinal chord. Where are we to find a clue out of this labyrinth of contradictions? A few years ago, cerebral pathology was regarded as a mass of contradiction, and by many it is still so estimated. I have always felt that it is not so; and although I may be unable clearly to see the unity, to descry the essential lesion, in these various details, still I am confident it is to be found, and I hope none of you will be deterred from research by these conflicting theories.

Let us proceed per viam exclusionis. You may rest assured, it is not chronic meningitis, in the ordinary sense of the term; it is not, (as I think) inflammation of the vesicular neurine; and we have known it to co-exist with softening of the medullary fibre, the opposite condition to that detailed by Foville. All observers will, I think, agree in locating the disease in the brain or spinal chord, notwithstanding Monsieur Lelut's cases; nor is it, as some whom I highly esteem imagine, dependent on the effused serum, so uniformly found in this malady, in the lateral ventricles of the brain, and beneath and within the sac of the arachnoid membrane; for we find this effusion in almost every chronic case of mania, and I have never seen a case of dementia without it, whether or not the mental affection was associated with general paralysis. The arrangements of the committee of this great institution have enabled me to see and examine the brains of fifty-five lunatics, who have died affected with general paralysis. I had examined them before I entered the Asylum, and my convictions from these facts are, that Foville has approached, perhaps realized, the truth, for he may have recognised other lesions than hardness, as the cause of general paralysis. (I have never read his paper on the subject, and am indebted to Dr. Prichard for the statement I have made respecting his conclusions.) If, however, he limits the cause to this special change, he is obviously wrong; the experience of Bayle, of Calmeil, of Rodriguez, of Lelut, and of others, proclaims that *softening* of this structure may co-exist with general paralysis, while Lelut (and I for one do not dispute his facts) shows that no lesion may be *perceptible.* Allow me to attempt an explanation of these discrepancies.

Faithfully have I recorded facts, as far as my feeble powers of observation have enabled me; and now that I am about to give my conclusions, I solicit a patient examination of them. Examine them thoroughly; contrast them with well-observed facts; and if, in your calm judgment, they are erroneous, discard them for ever. I dread to mislead you; and I now distinctly state, that what I am about to offer, is simply the result of my own ratiocination, to be accepted or rejected, as you may think fit. I speak to you as gentlemen, fitted by age, and education, and reflection for independent thought: and if I know myself, my only wish is to arrive at truth. I believe, then, that general paralysis may be produced *by any lesion* which involves in its action, and *partially* destroys, the whole plane of medullary fibres which comes into contact with the vesicular neurine of the periphery of the brain; that the same result would follow any lesion which *partially* changed the whole of the fibres of the pons Varolii, or the motory columns of the medulla oblongata. I have seen these structures in general paralysis, hard and shrunken, when the brain has been free from any other changes than those common to insanity uncomplicated with other maladies, and I have already drawn your attention to similar cases recorded by M. Rodriguez. Perhaps my language is not sufficiently explicit to convey a precise idea of my meaning, when I state, that in persons who become the subjects of general paralysis, there has been existing, for some time previously, a capillary hyperæmia of the brain, induced by intellectual exertions, or other causes; that, from some strong emotion, or from persistence in the aforesaid exertions, there is, from these capillaries, an outpouring of fibrinous fluid between the primary elements of the cerebral structure—this becomes coagulated, (or rather, partially organized,) and hence the augmentation, and partial induration, of the fibrous parts of the brain in some cases; in others, this blastema becomes converted into fibrine, and leads to great hardness, and a general contraction, or shrinking up, of the structure into which it has been effused. My opinion is, that this induration precedes the softening; in the great majority of cases; that being, in fact, an adventitious structure, it is deficient in vitality, and ultimately becomes soft and broken up; while in other instances, the blastema being poured forth from vessels in an anæmic condition, does not become organized, but remains permanently fluid, softening the histological elements around which it is poured, and producing what is generally understood as "Ramollissement" of the brain.

It does not militate against this idea, but, on the contrary, supports it, that general paralysis is frequently associated with diseases of the heart. It occurs for the most part in persons of a sensitive or impressionable nature, belonging to the nervo-lymphatic, or nervo-sanguineous temperaments—to a class of persons whom Hippocrates would consider as having a humoral diathesis, and in whom the "affections" had been strong and active. The apt comparison which has been made by our distinguished physician between this disease and the separate stages of intoxication, also shows that the nerve-fibre itself may be so far disturbed as to induce this malady without any appreciable structural change; in other words, that the blood may be so contaminated as to give rise to a temporary general paralysis; and if to a temporary disease, then to a permanent one; for it is obvious that the general paralysis would last precisely as long as the alcohol in definite quantities should remain in the blood unremoved by the excretory vessels of the system. Now, if it can be produced by one extraneous element in the blood, it may be by others; and hence the importance which a diseased heart, especially endocarditis, or an affected kidney, may have in inducing this malady, in such very rare and isolated cases as those detailed by Lelut, where no appreciable lesion could be detected in the brain or its membranes. This production of general paralysis by blood disease is not an idle hypothesis; epilepsy, and the disease called "delirium cum tremore," which is an evanescent type of this very formidable malady, has been proved to arise from the absorption of lead into the system. Dr. Todd, from whose profound researches and great acquirements we have learnt so much, respecting the physiology of the nervous system, has published the following interesting case:—

"A man, who had been several times the subject of lead colic, was brought to King's College Hospital, having had an epileptic fit; and being then in a state of coma; he soon had a second fit, after a few days he recovered considerably, but with much cerebral disturbance, and resembling a person in delirium tremens. Opium and other stimulants appeared to be beneficial, but after a few days he again became stupid, a fœtor of the breath indicated gangrene of the lung; after this he quickly sank. The brain was found to be pale, much shrunken, and the grey matter of a very light colour; a good quantity of sub-arachnoid fluid existed in consequence of the shrunken state of the brain. *There was no disorganization of the brain;* but on chemical analysis, there was found a great quantity of lead in the brain, and a still greater quantity in the lung, which was gangrenous in the centre."

Andral, Bouillaud, Bright, Macintosh, Watson, MacLeod, Hawkins, Burrows, and others, have given instances in which a diseased condition of the heart and pericardium have produced spasmodic diseases, general paralysis, mania, and even dementia; but for these particulars I refer you to the Sixty-first Lecture of Dr. Watson, to Copland's "Dictionary of Medicine," to Rostans upon the "Softening of the Brain;" but more especially to a very admirable treatise on the Cerebral Circulation, by Dr. Burrows, of St. Bartholomew's Hospital.

I wish, however, to add to these imperfect observations, that inflammation is a poor word to apply to those changes which produce general paralysis, inasmuch as by some special action upon the brain itself, the disease may be produced in a moment; for it is more frequently produced through the *emotions* than through the intellect. Last Christmas, I saw a gentleman who was suddenly paralysed by a piece of intelligence: there were all the symptoms of general paralysis in a most aggravated form; the quivering of the facial muscles on speaking, the tremor of the lips, the peculiar progression which has been described to you, and the quivering of the muscles after the slightest movement; there was a perfect loss of all power over the sphincters; and in three weeks he became altogether powerless, and in the fifth week he died.

This disease, then, in the great majority of cases, is one of impaired nutrition of the fibrous structure of the encephalon, and of some of the ganglia at the base of the brain, *commonly* induced by *intense* and long-continued intellectual exertions, *carried on under emotional excitement;* by some other causes, which seem to act specially on the nervous system itself; and occasionally from diseases of the heart, and other viscera, inducing sympathetic irritation of the above structures, or acting directly upon them by the poisonous elements which these respective diseases have contributed to the blood.

---

